# In Vivo Simultaneous Analysis of Gene Expression by Dual-Color Luciferases in *Caenorhabditis elegans*

**DOI:** 10.3390/ijms22010119

**Published:** 2020-12-24

**Authors:** Motomichi Doi, Megumi Sato, Yoshihiro Ohmiya

**Affiliations:** 1 Biomedical Research Institute, AIST, Tsukuba, Ibaraki 305-8566, Japan; satou-m@aist.go.jp (M.S.); y-ohmiya@aist.go.jp (Y.O.); 2 Department of Biomedical Engineering, Osaka Institute of Technology (OIT), Osaka, Osaka 535-8585, Japan; 3 Vidyasirimedhi Institute of Science and Technology (VISTEC), Reyong 21210, Thailand

**Keywords:** bioluminescence, gene expression, multi-color luciferases, *C. elegans*

## Abstract

Both fluorescent and luminescent observation are widely used to examine real-time gene expression patterns in living organisms. Several fluuorescent and luminescent proteins with specific optical properties have been developed and applied for simultaneous, multi-color observation of more than two gene expression profiles. Compared to fluorescent proteins, however, the application of multi-color luminescent imaging in living organisms is still limited. In this study, we introduced two-color luciferases into the soil nematode *C. elegans* and performed simultaneous analysis of two gene expression profiles. Using a green-emitting luciferase Eluc (emerald luciferase) and red-emitting luciferase SLR (stable luciferase red), the expression patterns of two genes were simultaneously observed in single animals from embryonic to adult stages over its whole life span. In addition, dual gene activities were observed at the single embryo level, with the simultaneous observation of morphological changes. These are the first application of a two-color luciferase system into a whole animal and suggest that precise relationship of expression patterns of multiple genes of interest can be analyzed over the whole life of the animal, dependent on the changes in genetic and/or environmental conditions.

## 1. Introduction

Measuring promoter activity of each gene is one of the most critical steps to understand molecular and genetic cascades involved in development and age-dependent regulation of life. In particular, analyzing the precise timing of on- and off-responses of promoters tells us how the activity of each gene is orchestrated, and how such interactions contribute to cellular and individual fates. For this purpose, in vivo imaging using luciferase reporters is widely used in various organisms. In addition, bioluminescent analysis is quantitative and applicable to high-throughput, automatic methods. Several luciferases have been isolated and developed to perform long-lasting time-lapse imaging, dual-or triple-gene imaging, and deep layer imaging in larger animals [1,2,3].

To date, we have established a multicolor reporter assay [4], multicolor real monitoring of gene expression [5], and bioluminescence imaging (BLI) at the single cell level [6] and at the subcellular level [7]. We have also established two color mice and demonstrated real-time monitoring of the expression of two genes in tissue culture samples [8]. Despite these developments of multicolor bioluminescence systems, simultaneous observation of multiple gene expression in whole organism during its whole life span is limited. In the human fungal pathogen *Candida albicans*, two luciferases from the click beetle were expressed and examined several promoter activities [9]. Two luciferases have been also expressed in higher plants [10] but not applied for multiple gene expression in the whole organism of higher animals.

*C. elegans* is a useful animal for image analyses not only by using fluorescent proteins but also luminescent proteins, due to its transparent and tiny body. Several recent studies showed that in vivo bioluminescent methods allowed the observation of developmental timing through its life stages [11], circadian-like periodic gene expression [12], and metabolic changes in cellular components [13]. However, simultaneous imaging using multiple luciferases to examine the activity of multiple genes (promoters) in vivo has not yet been performed. On the other hand, spatial-temporal gene expression patterns are widely examined using multiple fluorescent proteins. However, even if fluorescent proteins are expressed, their maturation times are relatively long (about 6 h), and their half-lives are also quite long (more than 24 h), compared to the short transition times for each developmental stage of *C. elegans*. Thus, fluorescent proteins can be used to easily estimate the onset timing of promoter activity but do not promptly indicate when activity goes down.

To examine the activities of multiple genes using luciferases in living *C. elegans*, we tested the *sur-5* and *myo-3* genes. The *sur-5* (suppressor of Ras) gene was first isolated as a suppressor of *let-60* Ras and shown to encode an acetoacetyl-CoA synthetase in worms [14]. The *sur-5* gene is expressed in most tissues of *C. elegans*, including the nervous system, hypodermis, body-wall muscles, and intestine, from the embryonic to adult stages. Furthermore, *sur-5* gene activity shows a periodical oscillation during larval to adult stages [12]. We also examined the *myo-3* gene, which encodes a myosin heavy chain isoform. This myosin heavy chain is strongly expressed in all muscle tissues [15,16]. In this study, we show that dual-color bioluminescent imaging in *C. elegans* enabled us to observe the expression profiles of two genes simultaneously, from the embryonic stage to the adult stage, at the single animal level.

## 2. Results

### 2.1. Real-Time Monitoring Two Promoter Activities in C. elegans

**Figure 1:**
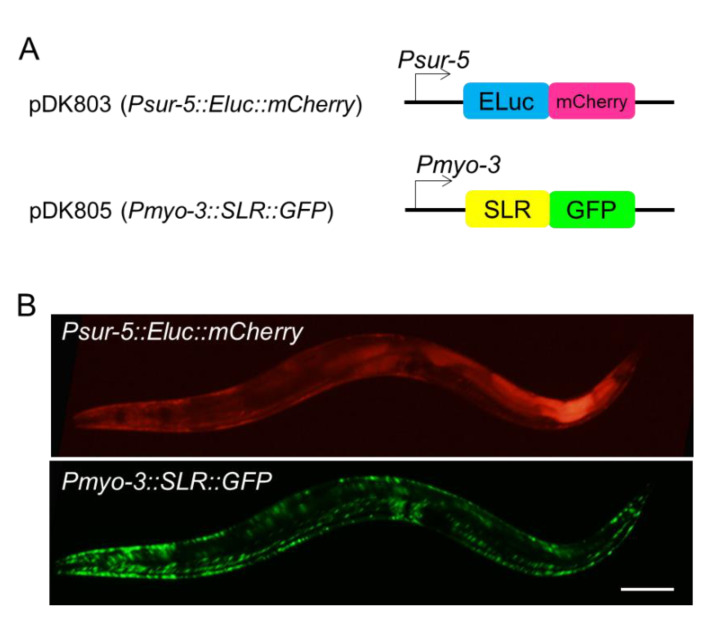
(**A**) Schematic representation of plasmid constructs for Eluc (Emerald Luciferase) and SLR (Stable Luciferase Red) expression. (**B**) Expression and localization patterns of Eluc (upper) and SLR (lower) fusion proteins in adult *C. elegans*. SLR protein tends to accumulate in body-wall muscles. Scale bar 50 μm.

To monitor two-gene expression profiles in living *C. elegans*, we simultaneously expressed a green-emitting luciferase Eluc (emerald luciferase) and a red-emitting luciferase SLR (stable luciferase red) in *C. elegans*. To know the expression and localization patterns of those luciferases in *C. elegans*, the Eluc protein was fused with the mCherry fluorescent protein and expressed in most tissues including the intestine, neurons, hypodermis, and muscles, under the control of the *sur-5* promoter. On the other hand, the SLR protein was expressed specifically in body-wall muscles, under the control of the *myo-3* promoter (Figure 1A). Expression and localization patterns of both fusion proteins were examined by observing fluorescent signals. Compared to the uniform localization of the Eluc-mCherry fusion protein in cells, the SLR-GFP (green fluorescent protein) fusion protein tended to accumulate in muscle cells (Figure 1B).

**Figure 2:**
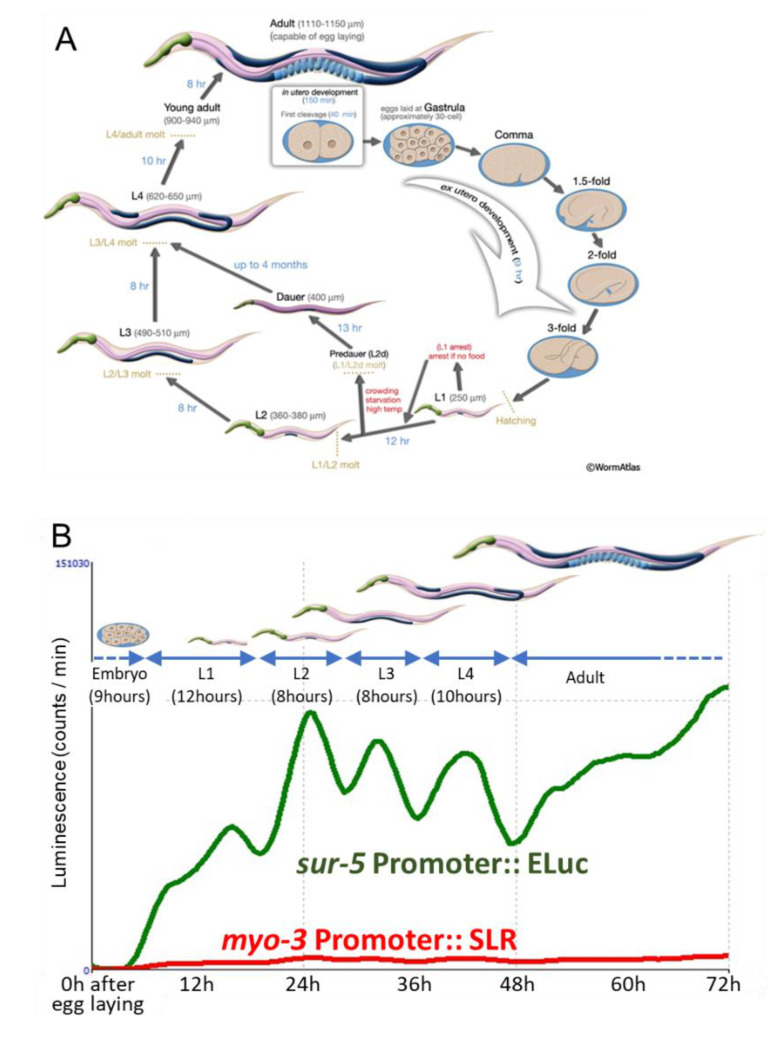
(**A**) The life cycle of *C. elegans* development (wormatlas; Altun, Z.F., Herndon, L.A., Wolkow, C.A., Crocker, C., Lints, R. and Hall, D.H. (ed.s) 2002–2020. http://www.wormatlas.org). (**B**) A representative trace of two luminescent signal changes. Pictures of each larval developmental stage are modified from A and shown above the trace.

**Figure 3:**
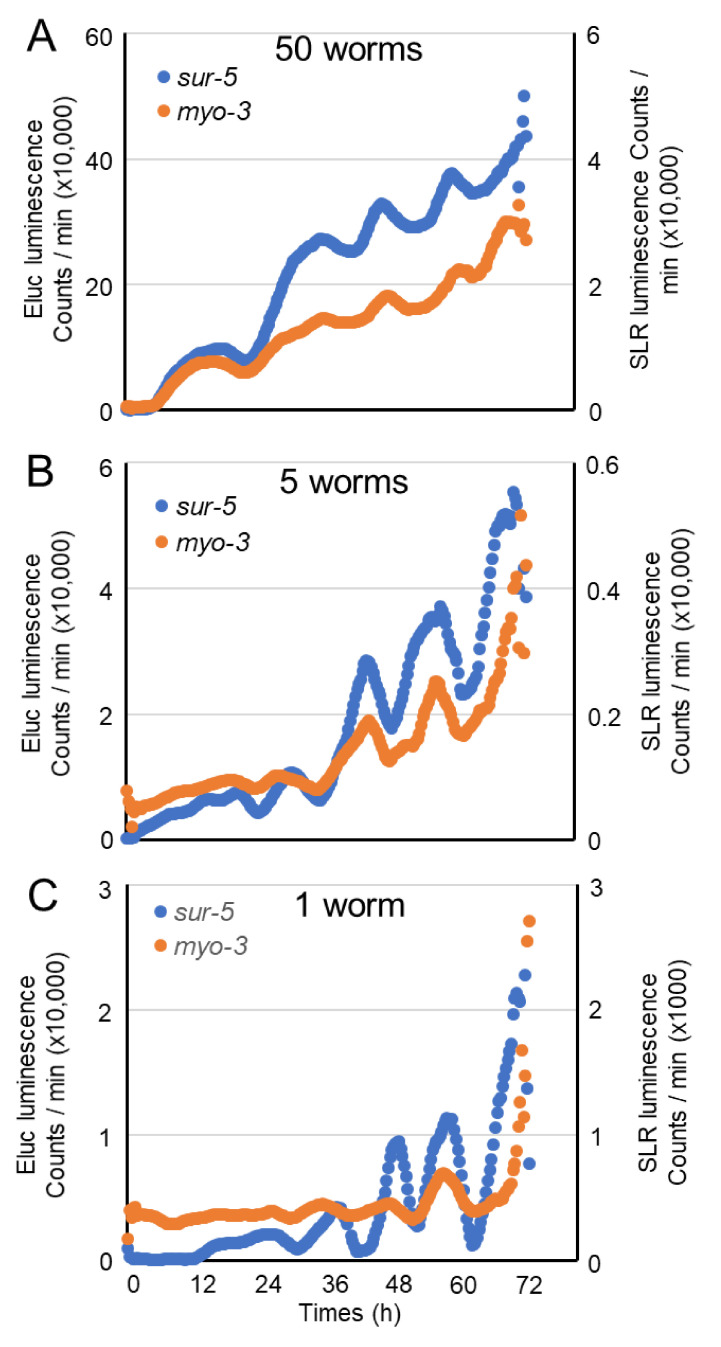
Representative traces of both Eluc and SLR luminescent changes during worm development in (**A**) fifty animals, (**B**) five animals and (**C**) a single animal. Left and right Y axes indicate luminescent counts for Eluc and SLR, respectively.

First, we examined whether the simultaneous, long-lasting observation of two-color luminescent signals can be performed by using the Kronos real-time monitoring system. We placed ~100 freshly laid embryos from animals expressing both Eluc-mCherry and SLR-GFP proteins into dishes and continued to observe them for up to four days. The sur-5 promoter activity was observed soon after the start of observation, and this Eluc signal showed up-and-down shifts lasting 8 to 12 h, suggesting that the periodical change in promoter activity may reflect the molting pattern of *C. elegans* (Figure 2A,B). Although the SLR signal did not show a dynamic signal change during the observation period due to the lower luminescent count rate from SLR, our results suggested that simultaneous observation would be possible using this system. Next, we set various numbers of embryos in each well and observed how many animals were sufficient to detect luminescent changes over three days. The observation of both fifty and five animals showed similar periodic fluctuation patterns in both *sur-5* and *myo-3* activities, but the baselines of both signals gradually increased (Figure 3A,B). We also observed clear promoter activities from single animals (Figure 3C). The periodical change in the Eluc signal, which corresponds to *sur-5* promoter activity was clearly monitored: the signal weakly fluctuated during the embryonic to L1 stages but was upregulated at the beginning periods of each larval stage. The signal was downregulated close to baseline presumably before molting. The *myo-3* activity monitored by the signal change in SLR also fluctuated through the developmental stages. However, the peak of *myo-3* expression in each larval stage seemed to be a few hours earlier than that of *sur-5*, especially in the L2 and L3 larval stages (Supplementary Figure S1A). Furthermore, its dynamics were much smaller than that of *sur-5* (Supplementary Figure S1B), suggesting that *myo-3* is not so dynamically up- and down-regulated through a worm’s life span. These results suggest that both *sur-5* and *myo-3* activities show similar periodic fluctuations dependent on the worm’s molting patterns, and that dual-monitoring of in vivo promoter activities is possible by using these two luminescent reporters. The periodical promoter activities in two genes was confirmed by using animals in which either Eluc or SLR protein (without fusing any fluorescent proteins) was expressed. Both *sur-5* and *myo-3* activities were periodically fluctuated, as seen in animals expressing both fusion proteins (Supplementary Figure S2). Although weak signal crosstalk may occur in luminescent signals, these data support that both promoters show similar periodical patterns of activity dependent on the development of worms.

### 2.2. Bioluminescent Imaging in the Developing Embryo of C. elegans

**Figure 4:**
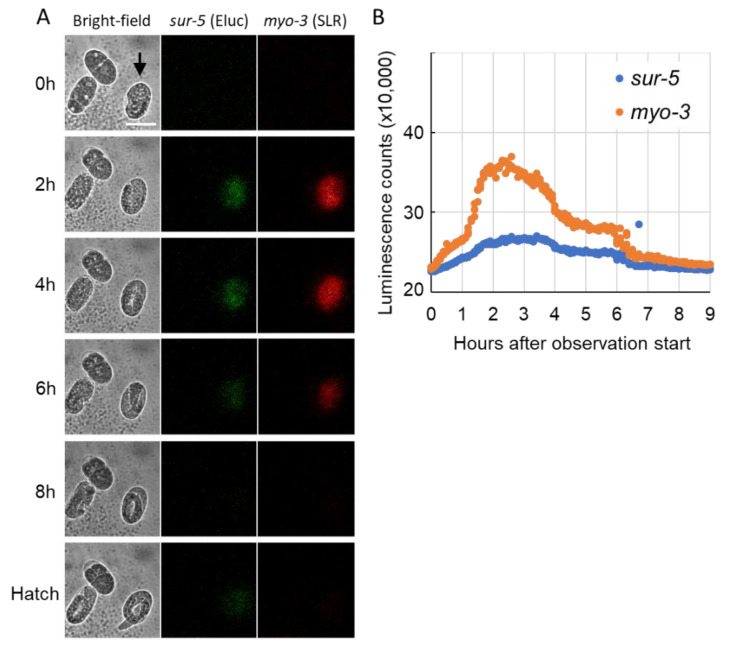
Simultaneous observation of multiple panromoter activities and morphogenesis in transgenic embryos expressing two luciferases. (**A**) Representative bioluminescent images of developing embryos. The bright-field image (left), green-channel image for Eluc (*sur-5* promoter activity, center) and red-channel image for SLR (*myo-3* promoter activity, right) are shown at selected time-points after the start of observation. (**B**) Luminescent count changes in a single embryo marked by an arrowhead in A. Scale bar 50 μm.

Analyses using a real-time monitoring luminometer showed that simultaneous monitoring of the activity of two promoters in single *C. elegans* animals is possible by using luminescent proteins, and that each promoter seemed to be dynamically regulated during each developmental stage. To ask whether the changes in luminescent signals are exactly correlated with the developmental timing of animals, we next tried to observe dual luminescent signal changes simultaneously with bright-field imaging by using a live-cell BLI system. However, we encountered several problems when monitoring promoter activities by BLI during *C. elegans* development: (1) more than ten seconds were required to acquire sufficient signals from a single animal, (2) during long time-lapse observation, most animals moved away from the field of view even if we used Unc mutant animals, and (3) inhibiting their movements by using glues disrupted molting. Due to these difficulties, we monitored promoter activities during embryogenesis. Embryos were collected from transgenic animals by dissection, put in a glass-bottom dish, and their luminescent signal changes were observed until the L1 hatching. In some embryos, both Eluc and SLR signals was unstable, for example, suddenly appeared at a late stage of embryogenesis such as the three-fold embryo and gradually decayed before the L1 hatching depending on the embryonic conditions (Supplementary Figure S3). However, in some embryos, significant luminescent signals gradually increased before the two-fold stage and had a peak until the three-fold stage of embryogenesis. Then, the signals gradually decayed before the L1 hatching (Figure 4). These data suggest that both *sur-5* and *myo-3* promoter activities peak a few hours before hatching and are then suspended until hatching. In addition, we found that luciferin can be introduced in developing embryos by unknown mechanisms, and that the real-time observation of gene expression in the *C. elegans* embryo can be performed with the regulation of luciferin introduction.

## 3. Discussion

In this study, we have shown that our luminescent system enables us to simultaneously monitor promoter activities of two genes. Not only over a long-lasting developmental life span, but also during single embryonic development, our strategy clearly revealed expression profiles of two genes. It is true that bioluminescent systems are widely used in many cells, tissues and organisms, but this is the first indication that two-luciferase observation can be applied over the whole life span of a single animal.

To monitor expression profiles along each developmental stage in *C. elegans*, a real-time monitoring luminometer system was used, and our method detected the expression profile in a single animal. At first, we had thought that several animals would be required to detect the two luminescent signals in worms. However, the signals from a single animal were sufficient to measure the expression profiles of genes of interest. Furthermore, single animal analysis indicated clear on and off responses of promoter activity along developmental stages. However, these responses of promoter activities seemed to be relatively slow (Figure 3C). One reason for the slow response could be due to the expression of fusion proteins with fluorescent proteins in the animals. The degradation speed of these fusion proteins may be slower than native luminescent proteins. However, the responses of promoter activities in animals expressing each luciferase were similarly slow (Supplementary Figure S2), suggesting that the fusion of fluorescent protein probably does not affect the response of luminescent signal. Previously, we had shown that a potent destabilization sequence from the Calpain 3 protein, which is quickly degraded within 10 min by autolysis, significantly increased the temporal response of Eluc luciferase in cultured cells [17]. By using such a destabilization sequence, the response of luminescent signals in *C. elegans* is likely improved, and we can estimate more precise gene expression profiles in animals. On the other hand, increasing the number of animals reduced the sharpness of the profile due to slight developmental timing differences in each animal. Although we have not examined the expression of other genes by using this method, we believe that our method is capable of monitoring the activity of two genes in a single animal. As shown in Figure 2B, the signal strength from the SLR red-emitting luminescent protein is quite low compared to the Eluc green one. The lower signal of the red luminescent protein could be resulted from the low sensitivity of photomultiplier detector in the real-time monitoring system. In fact, two luminescent signals in embryos were almost same level in the BLI system (Figure 4). In addition, this luminescent protein tends to accumulate in the cytoplasm of *C. elegans* cells by an unknown mechanism. This may be a first report about the distinct localization properties of two luciferase proteins in the whole animal. This aggregation may affect the signal strength from expressing tissues; however, it does not seem that the gene expression profile would be altered by this aggregation property of SLR. However, suitable selection of the appropriate luminescent protein used for promoters of interest will be required to obtain sufficient signals from lower numbers of target cells or smaller tissues.

The maturation and half-live times should also be cared while discussing the differences in each of promoter activity. The maturation time of Eluc and SLR in yeast is 39.8 and 16.7 min, respectively [18]. Comparing the peak times of *sur-5* and *myo-3* promoter activities in the 2nd larval stage, the *myo-3* activity becomes maximum much earlier than that of *sur-5* (31.7 vs. 34.9 h, Supplementary Figure S1A). Because this difference is significantly larger than the difference in maturation time, earlier promoter activity in *myo-3* did not result from the earlier maturation time of SLR. In case of other promoters whose activities change in much shorter duration, it could be due to the time differences in maturations of two luciferases. It is yet to be examined whether the maturation times of both luciferases in *C. elegans* are similar to those in yeast. Thus, additional data will be required to argue the precise timing of gene expressions in minutes level. We are not yet clear why the activity of *myo-3* is earlier than *sur-5* in L2 larval stage only. Further analyses may identify the detailed difference of myosin functions in each larval development.

As seen in the single animal analyses, luminescent observation clearly showed the on and off responses of promoter activity. Although a long-term, continuous observation of fluorescent signal from the same animal is technically hard in *C. elegans*, we performed a time-lapse fluorescent observation from the embryo to L2 stage animal. The GFP signal corresponding to *myo-3* promoter activity showed a slight fluctuated response similar to our luminescent analyses (Supplementary Figure S4). However, signals from some animals did not show clear on/off responses, and the red fluorescent signal (mCherry) was quite weak to perform a quantitative analysis (data not shown). A similar stable fluorescent signal from *sur-5* promoter activity between the L1 to L2 molt was observed in multi-well plate analyses [11]. Thus, to analyze the short-term fluctuating promoter activity seen in a *C. elegans* life stage, the luminescent approach provides better temporal resolution in the living organisms.

In addition, it is quite surprising that our method can illuminate two gene expression profiles in a single developing embryo, together with bright-field observation of morphogenesis. The *myo-3* promoter activity was shown to be active from the “pre-comma” stage of embryogenesis to L1 hatching, by both fluorescent marker analysis and transcriptome analysis [19,20], suggesting that our luciferase reporters may not be correct on time-points in embryos. However, our results firstly showed that both *sur-5* and *myo-3* promoters are downregulated before hatching. These fine resolutions of temporal gene activity have not been seen using other fluorescent protein analyses or traditional gene expression analyses. Although a technical modification for the luciferin introduction into the embryo must be developed, our multi-luciferase observation has strong potential to increase our understanding of the precise on and off timing of promoter activities.

In vivo observation of multiple gene expression patterns is possible for several organisms. *C. elegans* is a suitable animal for analyzing multiple gene expression profiles through the whole life cycle due to its small and transparent body and fast rate of development. These characteristics enable us to find novel gene expression profiles at specific timepoints in a selected animal. Single-animal analysis is required to elucidate how the activity of each gene is responsible for its characteristic features. We believe that our strategy will contribute to the identification of novel relationships between gene expression profiles and environmental stimuli, as well as individual variation of characteristics.

## 4. Materials and Methods

### 4.1. Strains

Worms were cultivated on standard NGM agar plates seeded with *E. coli* OP50 at room temperature (∼22 ℃). The *lin-15(n765)* mutant strain was used to generate transgenic animals, and the *unc-119(ed3)* mutant was crossed with transgenic animals to inhibit spontaneous worm locomotory activity during long time-lapse observations.

### 4.2. Molecular Biology and Transgenic Animals

Standard methods for molecular biology were used to construct plasmids. For the expression of green-emitting luciferase (Eluc), the mCherry coding sequence was first replaced with the eGFP sequence in the vector pPD95.75 (kind gift from Andy Fire). Then, the Eluc sequence was inserted at the 5′ site of the mCherry gene by an in-Fusion reaction to generate the Eluc-mCherry/pPD95.77 plasmid. For red-emitting luciferase (SLR) expression, the SLR sequence was directly fused with the eGFP sequence in pPD95.77, by using an in-Fusion reaction. The promoter sequences were inserted between the HindIII and KpnI sites of each resulting Eluc-mCherry/pPD95.77 or SLR-GFP/pPD95.77 plasmid. We used the following promoter regions for the developmental timing-specific expression: *sur-5* (1044 bp upstream from the ATG start codon) for many tissues, including the intestine, and *myo-3* (2400 bp) for body-wall muscles (Figure 1A). We have also generated fluorescent-protein-less plasmid DNAs by inserting each coding sequence of luminescent proteins under the same promoter sequences (P*sur-5*:: Eluc and P*myo-3*:: SLR).

To generate transgenic animals, the resulting plasmids were injected into *lin-15* mutant animals using a standard microinjection method [21]. An amount of 10 ng/L of each luciferase DNA and 50 ng/L of pbLH98 plasmid DNA was used as an injection marker.

### 4.3. Real-Time Monitoring of Gene Expression by Bioluminescent Imaging

Visualization of the activity of two genes in worms was performed using either a real time monitoring luminometer (Kronos Dio, ATTO, Tokyo, Japan) or live-cell BLI system (CellGraph, ATTO, Tokyo, Japan). Newly laid eggs (within one hour) were picked and put into 3.5 cm glass-bottom dishes with S-basal buffer containing both 1 mM luciferin (Wako, Tokyo, Japan) and 10 μM levamisole, which decreases worm locomotory activity. For monitoring of two genes from the embryo to adult lifecycle, each dish containing a fixed number of animals was set onto the holder and exposed for one minute to collect luminescence signals. Light signals were taken every 20 min for three days at room temperature, with 560 nm bandpass filter (for Eluc) or a 600 nm longpass filter (for SLR), respectively. For high-resolution single embryo imaging, the BLI system was set to capture both bright field and dual-color luminescent imaging. The time interval was set at 10 min, and the exposure times were set at 5 s for luminescent images and 0.2 s for bright field images, respectively. Both luminescent images were captured first, and around 10 s later, a bright-field image was captured, so a short time lag existed between luminescent and bright-field imaging. Eluc (green) and SLR (red) signals were observed by using a 507 nm bandpass and 620 nm longpass filters, respectively. For data analysis, an oval-shaped region of interest (ROI) was set to label the whole embryo, and the total luminescent signals were counted. Averaged background signal was subtracted in each image.

## Data Availability

The data presented in this study are available on request from the corresponding author.

## Supplementary Materials

The following are available online at https://www.mdpi.com/1422-0067/22/1/119/s1.
