# Supplementary material for: In Vivo Simultaneous Analysis of Gene Expression by Dual-Color Luciferases in Caenorhabditis elegans
Source: Int J Mol Sci. 2020 Dec 24;22(1):119. doi: 10.3390/ijms22010119 (PMC7795788; doi:10.3390/ijms22010119)

Supplementary figure S1. Quantitative analyses of gene expression profiles in single animals.

A

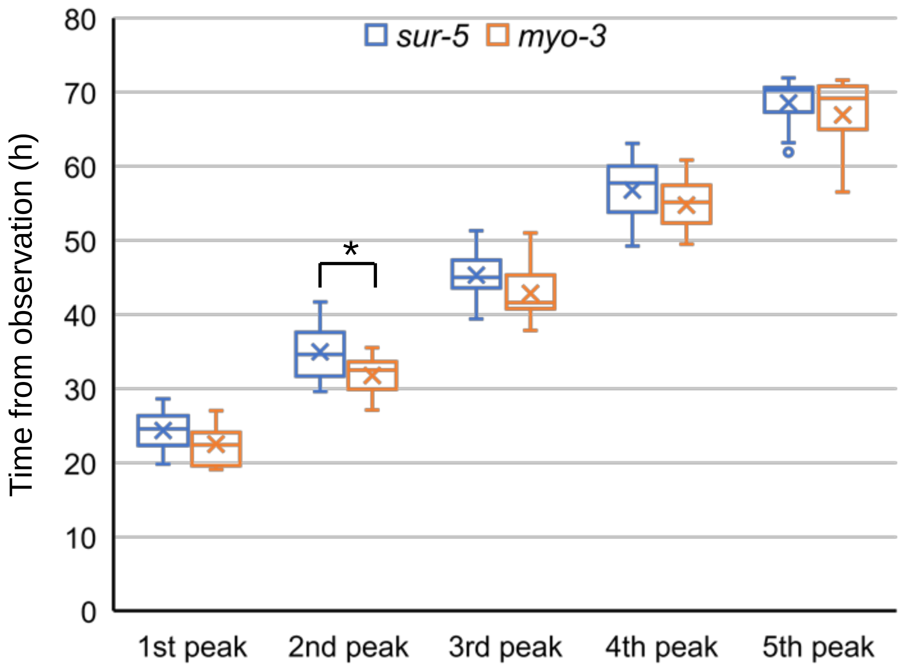

B

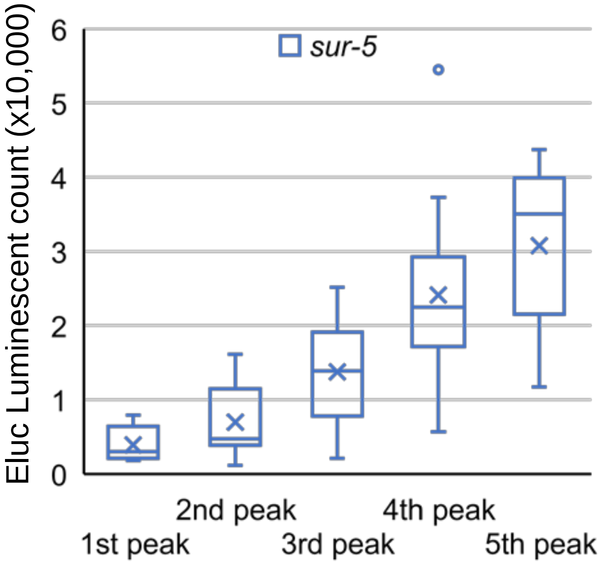

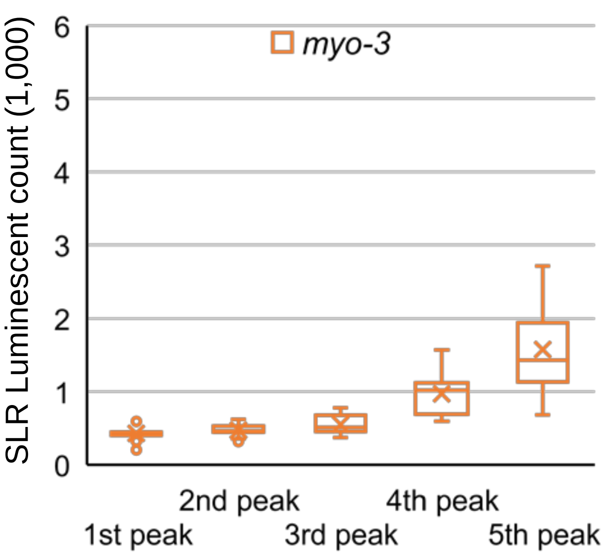

Supplementary figure S2. Luminescent signal changes in transgenic animals expressing single luciferase.

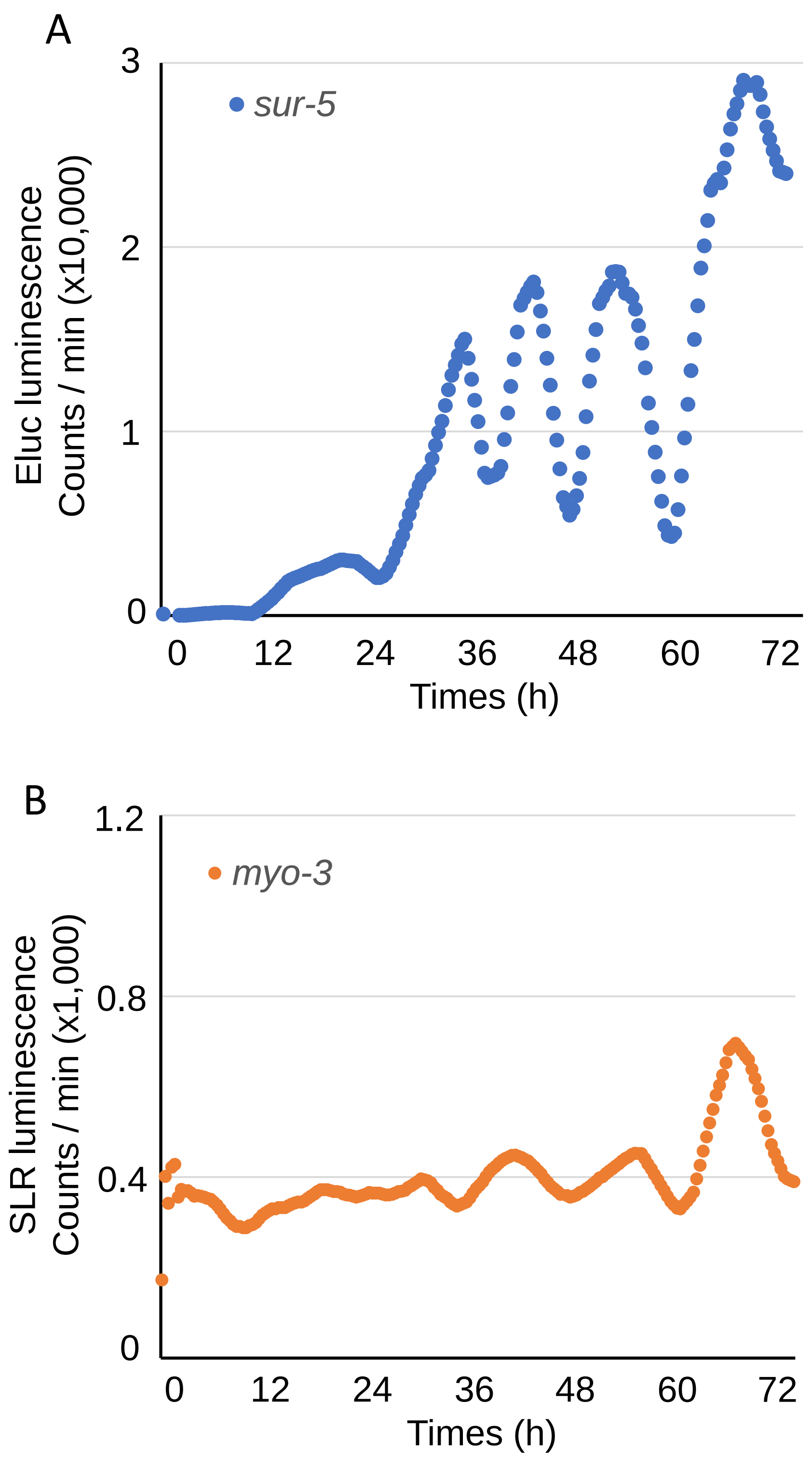

Supplementary figure S3. Examples of luminescent signal changes in transgenic embryos expressing two luciferases.

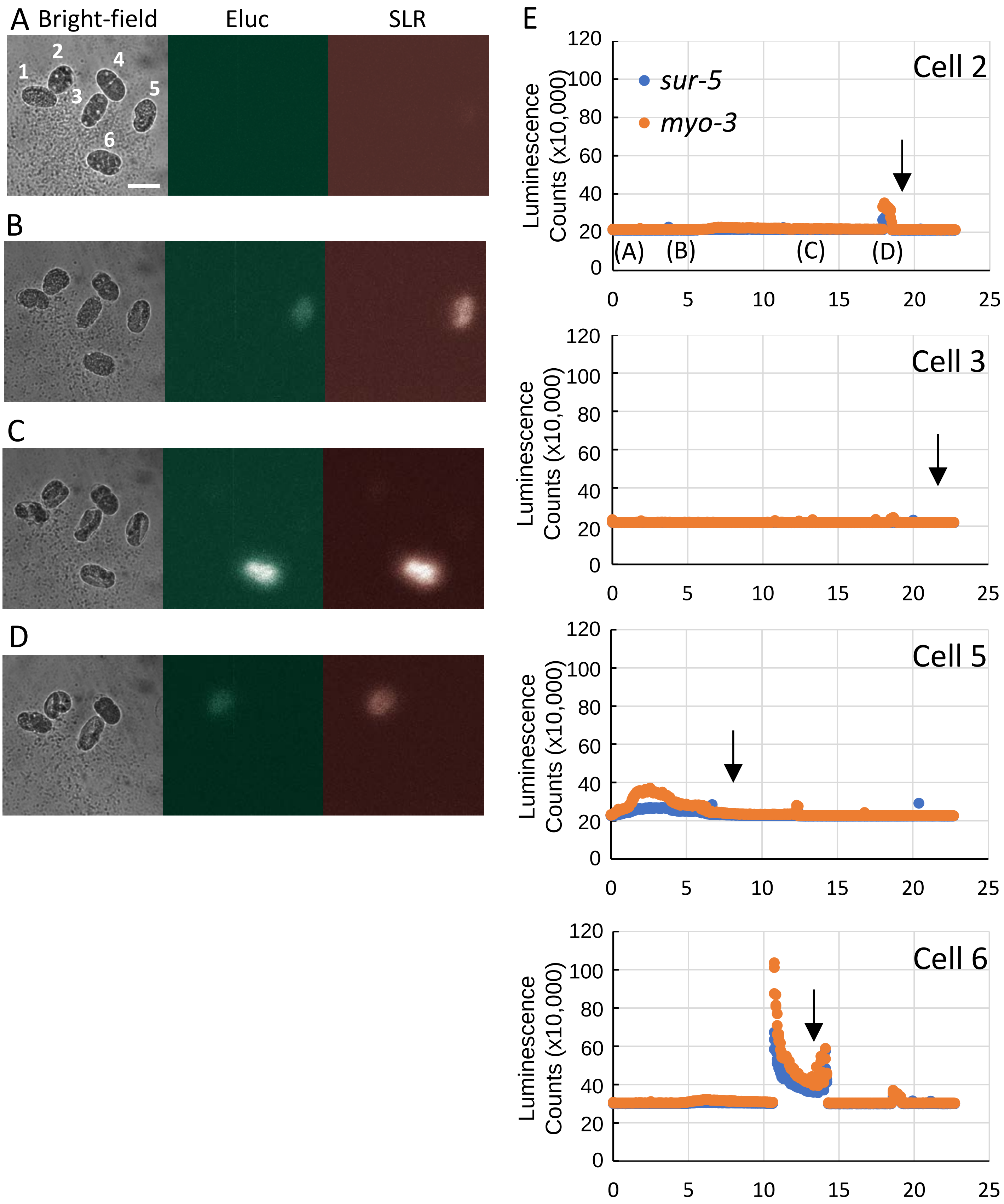

Supplementary figure S4. Time-lapse imaging of GFP signal change (myo-3 promoter activity) in the transgenic animals.

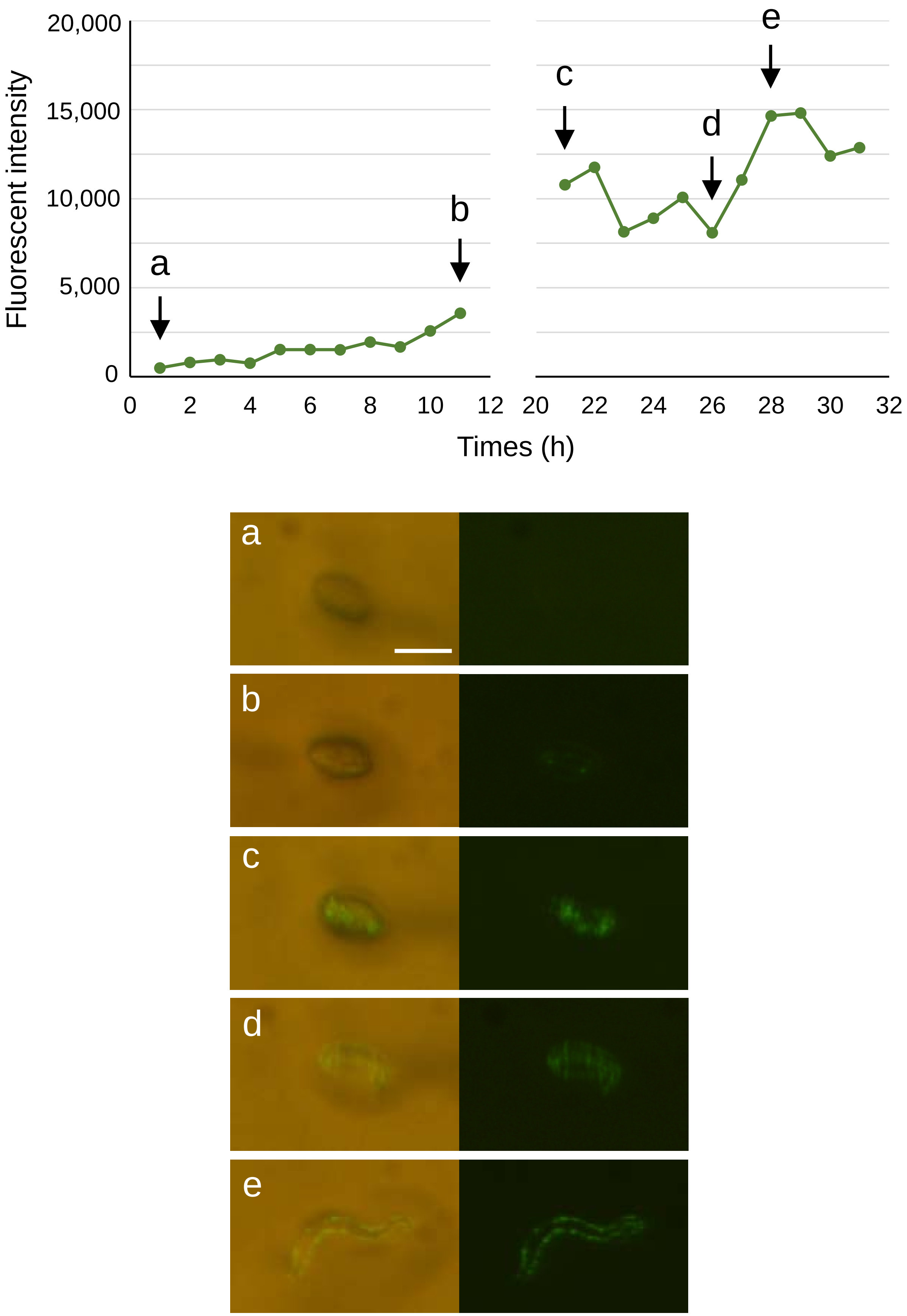

Supplement: Supplementary file 1 [file ijms-22-00119-s001.pdf]
